# Memantine and Cholinesterase Inhibitors: Complementary Mechanisms in the Treatment of Alzheimer’s Disease

**DOI:** 10.1007/s12640-013-9398-z

**Published:** 2013-05-09

**Authors:** Chris G. Parsons, Wojciech Danysz, Andrzej Dekundy, Irena Pulte

**Affiliations:** 1 In Vitro Pharmacology, Merz Pharmaceuticals GmbH, Eckenheimer Landstrasse 100, 60318 Frankfurt, Germany; 2 In Vivo Pharmacology, Merz Pharmaceuticals GmbH, Eckenheimer Landstrasse 100, 60318 Frankfurt, Germany; 3 Global Clinical Research and Development CNS, Merz Pharmaceuticals GmbH, Eckenheimer Landstrasse 100, 60318 Frankfurt, Germany; 4 In Vitro Pharmacology, Merz Pharmaceuticals GmbH, Eckenheimer Landstrasse 100, 60318 Frankfurt, Germany

**Keywords:** Acetylcholine, Alzheimer’s disease, Cholinesterase inhibitors, Glutamate, Mechanism of action, Memantine

## Abstract

This review describes the preclinical mechanisms that may underlie the increased therapeutic benefit of combination therapy—with the *N*-methyl-d-aspartate receptor antagonist, memantine, and an acetylcholinesterase inhibitor (AChEI)—for the treatment of Alzheimer’s disease (AD). Memantine, and the AChEIs target two different aspects of AD pathology. Both drug types have shown significant efficacy as monotherapies for the treatment of AD. Furthermore, clinical observations indicate that their complementary mechanisms offer superior benefit as combination therapy. Based on the available literature, the authors have considered the preclinical mechanisms that could underlie such a combined approach. Memantine addresses dysfunction in glutamatergic transmission, while the AChEIs serve to increase pathologically lowered levels of the neurotransmitter acetylcholine. In addition, preclinical studies have shown that memantine has neuroprotective effects, acting to prevent glutamatergic over-stimulation and the resulting neurotoxicity. Interrelations between the glutamatergic and cholinergic pathways in regions of the brain that control learning and memory mean that combination treatment has the potential for a complex influence on disease pathology. Moreover, studies in animal models have shown that the combined use of memantine and the AChEIs can produce greater improvements in measures of memory than either treatment alone. As an effective approach in the clinical setting, combination therapy with memantine and an AChEI has been a welcome advance for the treatment of patients with AD. Preclinical data have shown how these drugs act via two different, but interconnected, pathological pathways, and that their complementary activity may produce greater effects than either drug individually.

## Introduction

Alzheimer’s disease (AD) is the most common form of dementia. The prevalence of AD is strongly correlated with increasing age, and is a consequence of progressive neurodegeneration occurring over a period of several years, or even decades. This neurodegeneration leads to a gradual decline in cognitive, functional and behavioural processes, producing characteristic symptoms such as memory loss, confusion, agitation, and difficulties performing the activities of daily living. The symptoms become more severe over time, creating a decline in independence and an increasing reliance on caregiver support. By the end stages of AD, the patient is frequently bedridden, with substantial impairments (if not complete loss) of continence, swallowing, eating, speech, and motor abilities.

Extensive research into the underlying disease processes of AD has identified common pathological changes, with a leading hypothesis centring on neurotoxic protein deposits in the brain (amyloid plaques, neurofibrillary tangles). These are accompanied by disruptions in neurotransmitter levels, neuronal cell death, and brain (cortical) atrophy. Amyloid plaques consisting mainly of insoluble amyloid-beta peptide, and are a key histopathological feature of the AD brain. However, it has been proposed that the soluble oligomers of amyloid-beta, rather than the insoluble deposits, are primarily responsible for neurodegeneration and the impairment of synaptic function—including an effect on glutamatergic signalling pathways (see below) (De Felice et al. 2007; Lacor et al. 2007). Subsequently, pathological changes in neurotransmission can be associated with the characteristic clinical symptoms observed in AD. For example, in AD, there are impairments in glutamatergic and cholinergic signalling, which are involved in learning and memory processes in healthy individuals. Dysfunction in the glutamatergic system may lead to direct impairment of cognition and, in the long term, neuronal loss. Glutamate is responsible for approximately 70 % of the excitatory neurotransmission in the central nervous system (CNS), particularly in the cortical and hippocampal regions (Danysz et al. 2000). When the *N*-methyl-d-aspartate (NMDA) receptor is activated by glutamate, calcium (Ca^2+^) ions flow into the post-synaptic neurone (Dingledine et al. 1999). In a normal physiological situation, this triggers a signalling cascade that produces synaptic plasticity such as long-term potentiation (LTP) (Cacabelos et al. 1999), and thereby facilitates the higher order processes of learning and memory. According to some hypotheses, in AD, NMDA receptors are constantly over-activated, leading to sustained Ca^2+^ influx (Cacabelos et al. 1999; Danysz and Parsons 2003; Parsons et al. 1993). This creates a pathological ‘background noise’ against which normal physiological glutamate signalling cannot be detected (Danysz and Parsons 2003). Furthermore, prolonged Ca^2+^ overload in the post-synaptic neurones generates a form of ‘slow excitotoxicity’, producing a gradual neurodegenerative effect (Cacabelos et al. 1999; Danysz and Parsons 2003; Kornhuber and Weller 1997; Lancelot and Beal 1998; Parsons et al. 1998). It is probable that this pathological over-activity is due to increased resting glutamate concentrations, as well as increased receptor sensitivity to resting glutamate levels (Albin and Greenamyre 1992; Danysz and Parsons 2003). Certainly, some evidence suggests that various toxins, acting through NMDA receptors, e.g. amyloid-beta oligomers, can produce increased sensitivity and/or tonic activity of glutamate receptors, leading to neuronal death (De Felice et al. 2007; Lacor et al. 2007; Nyakas et al. 2011; Szegedi et al. 2005; Wenk et al. 2006; Wu et al. 1995) and decreasing synaptic plasticity (Nakagami and Oda 2002). In addition to this glutamatergic pathology, amyloid-beta peptides have also been shown to depress the release, synthesis, and axonal transport of acetylcholine (ACh) (Auld et al. 2002; Nyakas et al. 2011).

Cholinergic dysfunction in the basal and rostral forebrain is associated with even early cognitive impairments observed in AD, correlates with cognitive decline, and forms the basis of the ‘cholinergic hypothesis’ of AD (Perry et al. 1999; Terry and Buccafusco 2003). The involvement of the cholinergic system in AD was further strengthened by preclinical studies in which cholinergic antagonists were shown to impair memory in animals, and studies in which damage/lesions that interfered with cholinergic input from the basal forebrain (to the neocortex and hippocampus) led to memory deficits in both animals and humans (reviewed in Terry and Buccafusco 2003). In humans, several studies of scopolamine (a pan-muscarinic receptor antagonist) have indicated that cholinergic neurotransmission is pivotal to normal memory acquisition, facilitating immediate recall (Drachman 1977; Mewaldt and Ghoneim 1979; Petersen 1977)—possibly by preventing proactive interference between stored information and new memory (Atri et al. 2004). Furthermore, analyses of biopsy samples from the neocortex found that choline uptake, choline acetyltransferase activity and resulting ACh synthesis were all markedly reduced in patients with AD—indicating a loss of presynaptic cholinergic nerve endings (Sims et al. 1983). Overall, it is hypothesised that the degeneration of cholinergic neurones leads to a decline in the level of ACh, and the resulting weakened signalling contributes to cognitive decline and memory impairment in AD (Francis et al. 1985; Sims et al. 1983; Terry and Buccafusco 2003; Turrini et al. 2001). In addition, AD brains have a reduced number of cholinergic neuronal nicotinic receptor ion channels (α7 and α4β2) (Nordberg 2001) and functional uncoupling of cholinergic muscarinic M1 (G-protein coupled) receptors (Joseph et al. 1993; Thathiah and De Strooper 2009).

Reflecting these pathological patterns, the two drug classes currently available for the treatment of AD are the uncompetitive NMDA receptor antagonist, memantine (which normalises dysfunctional glutamatergic neurotransmission), and acetylcholinesterase inhibitors (AChEIs: donepezil, galanthamine and rivastigmine, which all raise ACh levels). As monotherapies, these drug types have demonstrated significant symptomatic efficacy in AD, and existing clinical data suggest that their combined use may bring additional benefit. For example, in a randomised, controlled clinical study of patients with moderate to severe AD, treatment with memantine and donepezil produced significant symptomatic (cognitive, functional, behavioural and global) advantages over donepezil monotherapy (Tariot et al. 2004). There are also indications that combined treatment could produce long-term benefits, and favourably influence time to nursing home admission (Atri et al. 2008; Lopez et al. 2009).

Here, we discuss the potential mechanisms that may underlie these clinical observations, including the scientific rationale for targeting two different neurotransmitter systems to achieve additive clinical effects.

### Mechanism of Action: Memantine

Memantine is indicated for the treatment of patients with moderate to severe AD, and has shown significant symptomatic efficacy in several large-scale, controlled clinical studies (Reisberg et al. 2003; Tariot et al. 2004; Winblad et al. 2007). There is also evidence that memantine may be effective in delaying clinical worsening, and decreasing the emergence of behavioural symptoms including agitation and aggression (Wilcock et al. 2008; Wilkinson and Andersen 2007).

Memantine has been characterised as an uncompetitive, voltage-dependent NMDA receptor antagonist, with moderate binding affinity, and rapid blocking–unblocking receptor kinetics (Danysz et al. 2000; Parsons et al. 1993). This unique binding profile enables memantine to integrate in the glutamatergic signalling system, and influence dysfunctional NMDA receptor activation in AD. Under normal, resting, physiological conditions, NMDA receptor channels are blocked by magnesium (Mg^2+^) ions. Upon the arrival of a strong transient glutamate synaptic signal, the post-synaptic membrane becomes depolarised, relieving the voltage-dependent Mg^2+^ channel block, and the NMDA channel opens to allow Ca^2+^ ion flow into the post-synaptic neurone. However, it has been suggested that in AD, due to glutamate and amyloid-beta driven constant low-level stimulation there is a decrease in membrane potential, removing the NMDA receptor channel blockade by Mg^2+^ ions, increasing the continuous flow of Ca^2+^ ions into the post-synaptic neurone, and thereby creating a ‘background noise’ of stimulation at rest. Electrophysiological studies have indicated that memantine binds within the ion channel of the NMDA receptor (Chen et al. 1992; Parsons et al. 1993). Importantly, memantine binds with moderate affinity and voltage dependency, which means that at moderate levels of prolonged stimulation (i.e. under pathological conditions) memantine continues to block the NMDA receptor channel—unlike the weaker binding Mg^2+^ ion (Albrecht et al. 2008; Parsons et al. 1993, 1999a, 2007). However, when high concentrations of glutamate are transiently present (i.e. when a physiological signal arrives), memantine dissociates from the receptor, and normal neurotransmission proceeds (Albrecht et al. 2008; Frankiewicz and Parsons 1999; Parsons et al. 1999a, 2007). Thus, in AD, memantine may reduce the pathological ‘background noise’ of dysfunctional glutamate signalling, allowing physiological signals to be better distinguished (Danysz et al. 2000). Furthermore, in vivo and in vitro testing has shown that this effect translates into a reversal of learning impairments induced by over-activation of NMDA receptors (e.g. Zajaczkowski et al. 1997).

In addition to this facilitation of signalling processes, preclinical investigations indicate that memantine’s mechanism may also serve to protect neurones from the excitotoxicity of excessive glutamate stimulation. This has been demonstrated in vitro (Parsons et al. 1999a, b), and in vivo where memantine protected against excitotoxicity induced by direct or indirect over-activation of NMDA receptors (Keilhoff and Wolf 1992; Misztal et al. 1996; Wenk et al. 1994, 1995; Willard et al. 2000).

As mentioned earlier, the presence of amyloid-beta has been linked with pathological effects on the glutamatergic system, indirectly triggering excess tonic levels of glutamate in and around the synaptic cleft by inhibiting the astroglial glutamate transporter (Nyakas et al. 2011) and reducing glutamate reuptake (Harris et al. 1996; Noda et al. 1999). According to some authors, amyloid-beta peptides may also stimulate NMDA receptors either as a direct agonist, or secondary to interactions with, e.g. post-synaptic anchoring proteins (De Felice et al. 2007; Mattson et al. 1993; Szegedi et al. 2005; Wu et al. 1995).

Linking in with the ‘amyloid-beta hypothesis’, memantine has been shown to protect against pathological changes and learning deterioration induced by intra-hippocampal injection of amyloid-beta (Miguel-Hidalgo et al. 2002). Moreover, memantine restored deficits in cognition, and reduced levels of insoluble and soluble amyloid-beta peptide in triple-transgenic mice with AD-like pathology (Martinez-Coria et al. 2010). Memantine has also specifically demonstrated protection against amyloid-beta-induced synaptic deterioration (Lacor et al. 2007) and generation of reactive oxygen species (De Felice et al. 2007; Klein et al. 2007). Despite these findings the way in which memantine’s mechanisms link to its clinical efficacy in AD has still not been fully characterised.

### Mechanism of Action: Acetylcholinesterase Inhibitors

The AChEIs (donepezil, galanthamine and rivastigmine) are indicated for the treatment of AD from the mild stages onwards (Birks 2009; Wilkinson et al. 2004). By inhibiting the action of the ACh-hydrolyzing enzyme acetylcholinesterase (AChE—the predominant cholinesterase in the brain), the AChEIs aim to boost ACh levels and thus alleviate disease symptoms associated with the progressive loss of cholinergic function in AD. Studies have shown that lowered synthesis of ACh (including reduced choline acetyltransferase activity, required for ACh synthesis) is associated with greater cognitive impairment in dementia, including AD (Francis et al. 1985; Perry et al. 1978). In contrast, raised ACh concentrations in the brain have been shown to increase the expression of nicotinic ACh receptors on cholinoceptive neurones (Barnes et al. 2000), and are also linked to improved function of other neurotransmitter systems associated with cognitive function, e.g. glutamate (Dijk et al. 1995; Francis et al. 1993). Therefore, AChE inhibition was identified as a useful therapeutic strategy to enhance cholinergic neurotransmission, even though patients with advanced AD show reductions in AChE levels of up to 90 % (Giacobini 2003). Clinical support for this treatment approach comes from multiple clinical studies in which AChEIs have benefited patients’ cognitive, functional and global status (Birks 2009). However, despite clear evidence of efficacy in the clinical setting, the exact mechanisms and pathways that link AChE inhibition and ACh activity with symptomatic improvements in AD are not fully understood.

Although the three AChEIs display no notable differences in their clinical efficacy (Birks 2009), their underlying mechanisms can be distinguished in terms of target protein specificity. Donepezil is a specific and reversible inhibitor of AChE (Davidsson et al. 2001; Wilkinson et al. 2004), and also independently interacts with neuronal nicotinic ACh receptors (Di Angelantonio et al. 2004). In contrast, rivastigmine is a pseudo-irreversible inhibitor of AChE (Davidsson et al. 2001; Weinstock 1999) (i.e. via a temporary covalent bond), and has a similar level of affinity for butyrylcholinesterase (BuChE) (Weinstock 1999; Wilkinson et al. 2004). BuChE is a non-specific enzyme that can hydrolyse ACh and other choline esters, and which is most predominant outside the CNS, with brain levels increasing in severe AD (Giacobini et al. 1989; Weinstock 1999; Wilkinson et al. 2004). Galanthamine is a selective, reversible inhibitor of AChE and is claimed to enhance the intrinsic action of ACh on nicotinic receptors, with a potential link to amyloid-beta clearance (Popa et al. 2006; Takata et al. 2010; Wilkinson et al. 2004)—although this latter effect is controversial. It does not seem that these different specificities contribute substantially to any differentiation in the therapeutic effects of the AChEIs (Wattmo et al. 2011).

### Combining the Action of Memantine and the AChEIs

As described above, memantine and the AChEIs target two different pathological aspects of AD—the dysfunctional glutamatergic and cholinergic transmitter systems, respectively. The combined use of memantine and an AChEI in the treatment of AD is therefore a logical, rational approach, although the underlying mechanisms and interactions are likely to be complex and partially reciprocal as their signalling pathways are interconnected and multiplexed.

#### Connections in the Glutamatergic and Cholinergic Pathways

**Fig. 1:**
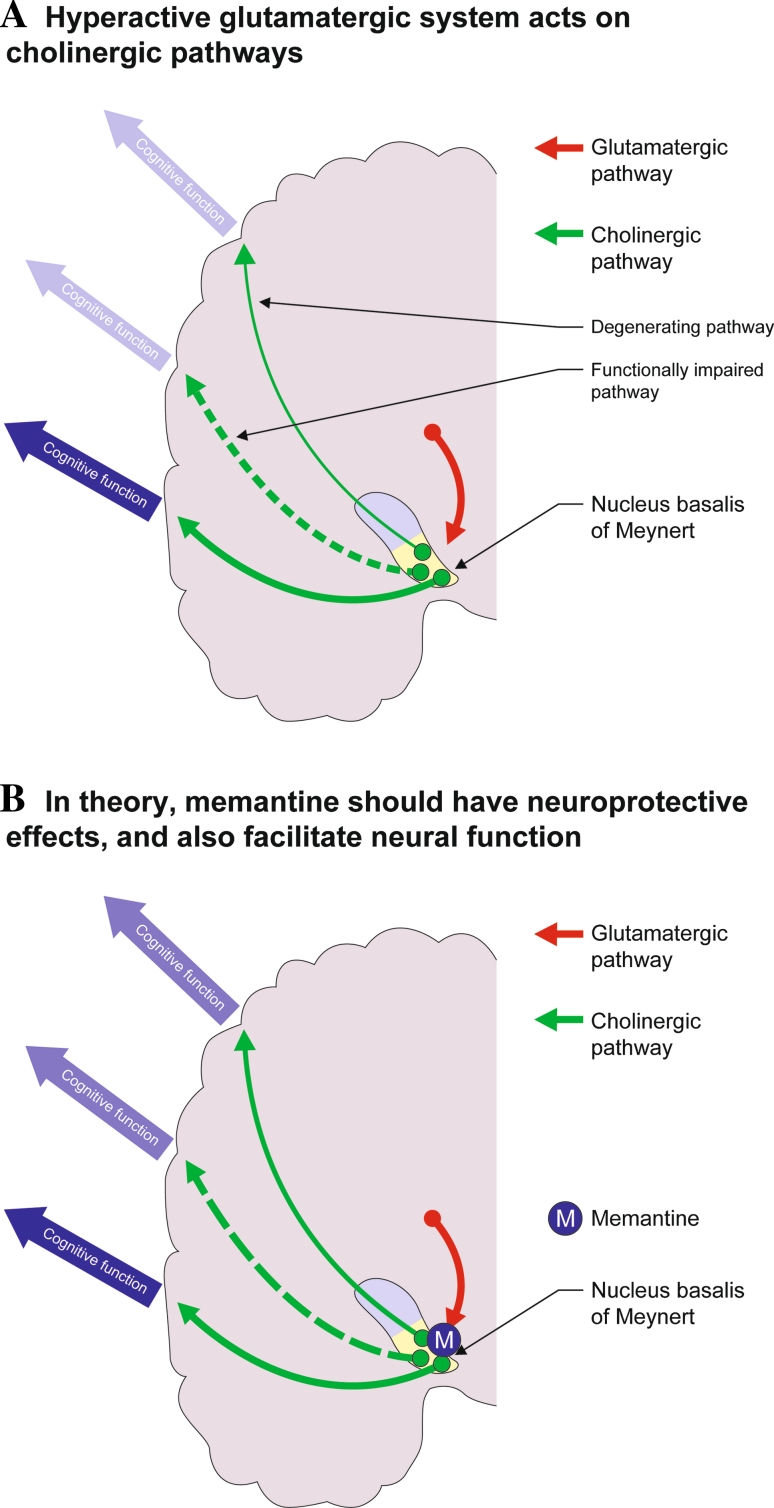
Links between the glutamatergic and cholinergic pathways

There is considerable evidence supporting the link between the cholinergic and glutamatergic pathways, including their relationship in the pathology of AD. Glutamatergic neurones in the amygdala, reticular formation, hippocampus and cerebral cortex make synaptic connections with cholinergic neurones located in the medial septum, diagonal band of Broca and the nucleus basalis of Meynert (NbM). These cholinergic neurones, in turn, innervate the neocortex and hippocampus (Fig. 1a) (Bigl et al. 1982; Carnes et al. 1990; Fadel et al. 2001; Fournier et al. 2004; Francis et al. 1985, 1993; Mesulam and Mufson 1984; Moor et al. 1996; Wu et al. 2004). There is also evidence that synaptic activation of NMDA receptors stimulates synaptic ACh release from basal forebrain neurones (Fournier et al. 2004; Moor et al. 1996), although these findings are somewhat controversial (Fadel et al. 2001; Giovannini et al. 1994). In addition, some ‘cholinergic’ neurones of the basal forebrain appear to release both ACh and glutamate (Allen et al. 2006).

Enhancement of ACh transmission and/or activation of nicotinic/muscarinic acetylcholine receptors increases LTP in most regions of the hippocampus, where glutamatergic pyramidal neurones provide the major intrinsic projection pathways (Drever et al. 2011; Kenney and Gould 2008). NMDA, amyloid-beta, and a decrease in Mg^2+^ ions, all impair LTP in the hippocampus; an effect that is prevented by the action of memantine (Frankiewicz and Parsons 1999; Klyubin et al. 2011; Martinez-Coria et al. 2010; Parsons et al. 1999a).

In terms of neurodegeneration, excessive activation of glutamate receptors (particularly NMDA-type receptors) has been implicated in processes underlying the degeneration of cholinergic cells in AD (Greenamyre and Young 1989). For example, neuronal degeneration caused by direct injection of NMDA into the rat basal forebrain leads to reduced levels of choline acetyltransferase activity in the cortex, and this effect is prevented by memantine (Wenk et al. 1994, 1995). Furthermore, memantine rescues basal forebrain cholinergic neurones from the toxic effects of amyloid-beta peptides, and attenuates amyloid-beta-induced cholinergic fibre loss in the parietal neocortex (Fig. 1b) (Nyakas et al. 2011).

Therefore, it seems that cholinergic neurones are vulnerable to the hyperactivity of the glutamate/NMDA receptor system, while glutamatergic neurones may be both the cause and the subsequent target of neuronal degeneration.

#### Preclinical Data on the Combined Action of Memantine and the AChEIs

In light of the interplay between the glutamatergic and cholinergic pathways, the combined use of memantine and the AChEIs has been examined in several preclinical studies. Importantly, investigations in vitro, in vivo and ex vivo have shown that memantine does not attenuate the AChE blockade produced by therapeutically relevant concentrations of clinically used AChEIs (Enz and Gentsch 2004; Gupta and Dekundy 2005; Wenk et al. 2000).

With regard to combined efficacy, the effect of memantine plus donepezil on cognitive deficits was examined in triple-transgenic mice exhibiting cognitive impairment and high brain levels of amyloid-beta plaques and neurofibrillary tangles (Martinez-Coria et al. 2009). In 6- and 15-month-old transgenic mice, memantine, and the combination of donepezil and memantine, significantly improved spatial memory (acquisition and retention) (Martinez-Coria et al. 2009). Donepezil treatment alone significantly improved the retention (but not the acquisition) of spatial memory in younger mice, but no significant effects were seen in older mice (Martinez-Coria et al. 2009). In both age groups, the memantine–donepezil combination was the only treatment that significantly improved latency to reaching the platform location (Martinez-Coria et al. 2009).

The combined effects of memantine and donepezil on spatial memory were also specifically examined in the APP23 mouse model using a complex dry-land maze test (Neumeister and Riepe 2012). In 4.5-month-old APP23 mice with cognitive deficits, treatment with memantine plus donepezil produced significant improvements in both resting time and moving time, which were greater than those observed with either treatment alone (Neumeister and Riepe 2012). Notably, treatment with memantine alone produced a significant improvement in resting time, but not moving time, and donepezil treatment improved moving time, but not resting time (Neumeister and Riepe 2012). These findings led the authors to suggest that combination treatment with memantine and donepezil was exerting a synergistic effect on spatial learning ability in these animals, with the individual drugs acting differentially: memantine addressing resting time (possibly reflecting memory retrieval), and donepezil influencing moving time (possibly reflecting memory acquisition) (Neumeister and Riepe 2012).

#### 3-Neurone Model

Based on available preclinical evidence of the actions of memantine and the AChEIs, the 3-neurone model is proposed to explain the better efficacy of combination treatment.

**Fig. 2:**
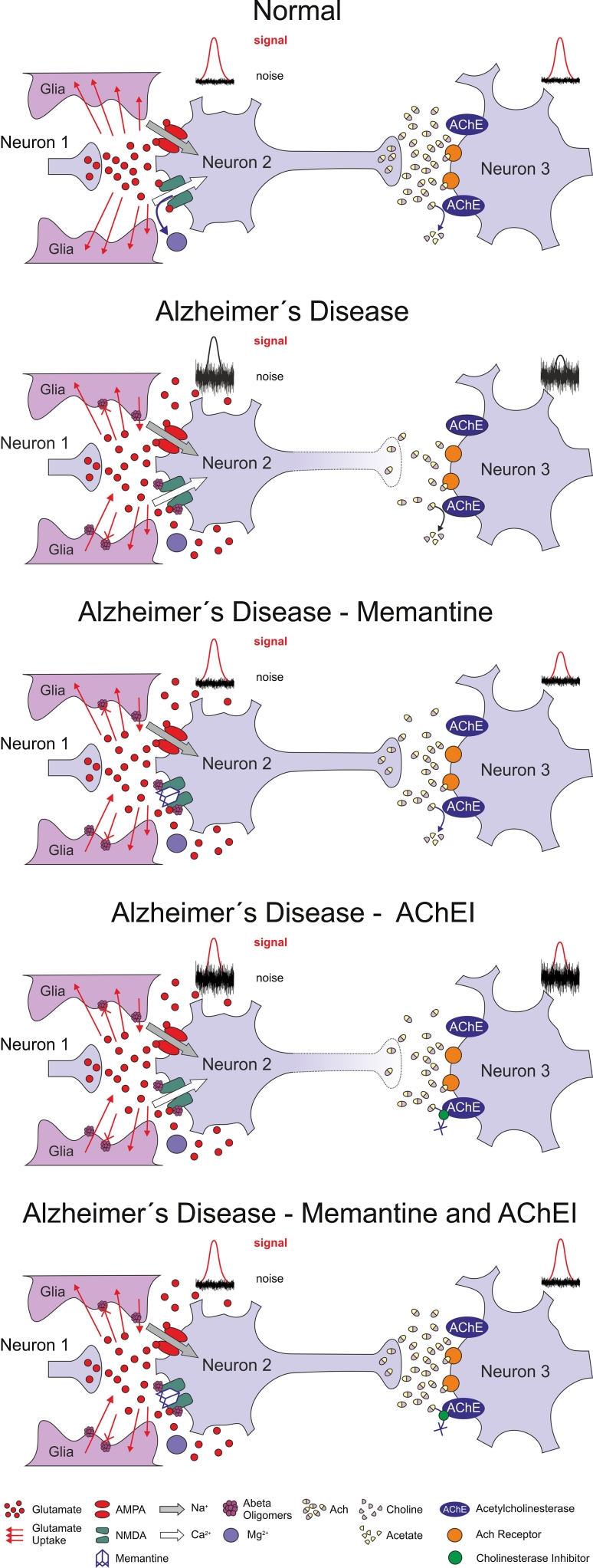
The ‘3-neurone model’ for the action of memantine and the AChEIs in AD

**Fig. 3:**
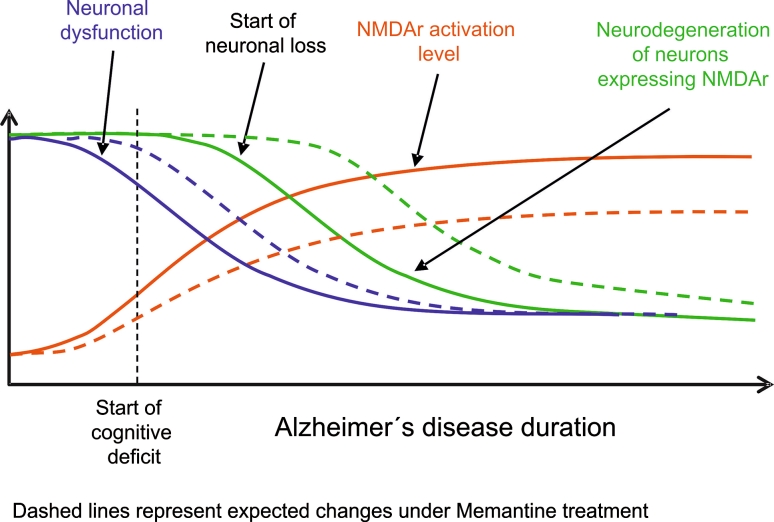
Schematic showing the proposed effect of memantine on the activity of NMDA

It is established that memantine and the AChEIs intervene at separate points of the disrupted signalling cascades in AD. Acting at the NMDA receptor, memantine lowers the pathologically increased tonic level of excitation of the glutamatergic synapse at rest (neurone 2—see Fig. 2). This is likely to have a twofold impact: firstly, it reduces the background noise, so that incoming physiological signals can be better distinguished; secondly, it reduces the constant pathological influx of Ca^2+^, and thereby helps to prevent the neurone being stimulated in a way that would cause both dysfunction, synaptotoxicity and ultimately cell death (neurone 2) (Parsons et al. 1999a, 2007). Overall, tonic NMDA receptor activation is reduced, which delays the neurodegeneration of cholinergic neurones bearing NMDA receptors, and synaptic NMDA receptor activation is facilitated (Fig. 3). Supplementing this effect, the AChEIs may serve to amplify (i.e. bring towards normal) the pathologically weakened signal from cholinergic neurones by delaying ACh breakdown at cholinergic nerve endings (reviewed in Palmer and Gershon 1990). In this way, neurotransmission (to neurone 3) is preserved, with the improved signal detected against the lowered background noise (Fig. 2). Together, such effects would help to maintain the glutamatergic/cholinergic signalling cascades, and consequently facilitate LTP and memory processes.

Clearly, this model is an integrated and simplified view of what is known to be a highly complex system. In particular, the 3-neurone model does not take into account that neurotransmission is unlikely to be a simple one-directional process. In practice, glutamatergic neurones not only make synaptic connections with cholinergic neurones, but cholinergic neurones also influence glutamatergic transmission in areas such as the cortex and hippocampus. Therefore, applied together, memantine and AChEIs have the potential to act at different places in interconnected pathways, with complementary mechanisms potentially producing additive effects opposing disease pathology. In addition to this restoration of function, memantine also appears to protect against excitotoxicity and therefore consequent neurodegeneration.

### Clinical Implications

**Fig. 4:**
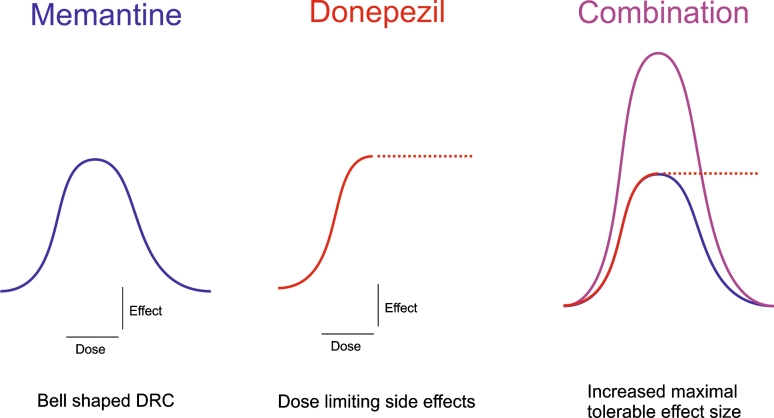
Schematic illustration of the hypothesised clinical effect size with combined memantine and AChEI treatment

It is well documented that the glutamatergic and cholinergic neuronal systems influence each other, and that their joint dysfunction is central to the effects produced by AD pathology. Consequently, the combined use of memantine and an AChEI to address these two pathological aspects of AD would appear to be a highly rational approach to treatment. From a pharmacodynamic perspective, combining two different drug mechanisms is a recognised means of increasing overall effect size, via a synergistic or additive interaction. This is schematically illustrated in Fig. 4, which shows how combining memantine and the AChEIs could produce a greater clinical effect size without exceeding the individual dose-limiting boundaries for either drug. Beyond this, preclinical studies also indicate that memantine may confer a neuroprotective benefit.

It is interesting to note that in monotherapy, memantine and the AChEIs appear to have additional positive effects on some differing behavioural domains—memantine producing improvements in agitation/aggression and delusions (Gauthier et al. 2005), and AChEIs (donepezil) affecting the domains of depression, anxiety and apathy (Feldman et al. 2001). This would seem to be further evidence (albeit indirect) that the two drugs do not act via the same mechanisms, or exclusively within the same pathways/brain regions.

In practice, the application of combination treatment in AD obviously relies upon proven safety and efficacy in the clinical setting. Drug interaction studies in healthy adults (Periclou et al. 2004; Yao et al. 2005) and in patients with AD (Shua-Haim et al. 2008) have shown a lack of pharmacokinetic interactions between memantine and each of the AChEIs. Combination treatment has also proven to be well tolerated in randomised, controlled studies of patients at all stages of AD (Porsteinsson et al. 2008; Tariot et al. 2004; Grossberg et al. 2008; Howard et al. 2012), as well as in post-marketing surveillance of dementia patients in German clinical practice (Hartmann and Möbius 2003). Indeed, some gastrointestinal side effects of the AChEIs (diarrhoea, faecal incontinence, nausea) have been reported less frequently with combination treatment (Tariot et al. 2004) and discontinuation rates after 24 weeks are also decreased compared with AChEI monotherapy (Porsteinsson et al. 2008; Tariot et al. 2004). The pooled analysis of two randomised, controlled 24-week studies has shown a similar incidence of adverse events in patients treated with the combination of memantine and donepezil and in patients treated with placebo and donepezil (Atri et al. 2013). Interestingly, this pooled analysis, performed in patients with moderate to severe AD, has also shown that the frequency of agitation was approximately two times less in the memantine/donepezil combination treatment group compared to placebo/donepezil group.

In terms of efficacy, several large-scale clinical studies have included patients receiving combination treatment, and shown favourable outcomes (Tariot et al. 2004; Atri et al. 2008; Lopez et al. 2009; Grossberg et al. 2008). In one randomised, double-blind controlled study in 404 patients with moderate to severe AD, treatment with memantine and donepezil produced significant benefits across cognitive, functional, behavioural and global domains, when compared with donepezil monotherapy (Tariot et al. 2004). Post hoc analyses of this study revealed the superiority of combination treatment over AChEI monotherapy in specific areas such as language and memory (Schmitt et al. 2006), toileting and higher-level functions (Feldman et al. 2006), and agitation/aggression and irritability (including decreased symptom emergence) (Cummings et al. 2006; Gauthier et al. 2005). In addition, more patients responded with symptom improvement or stabilisation in the combination treatment group than in the group receiving monotherapy with donepezil (van Dyck et al. 2006).

Different results were reported when memantine/AChEI treatment was investigated in a randomised, double-blind controlled study in 433 patients with mild to moderate AD, in which the combination was not found to be statistically superior to AChEI monotherapy (Porsteinsson et al. 2008). The apparently contradictory results from these two studies (Tariot et al. 2004; Porsteinsson et al. 2008) have led to some uncertainties in recommendation of the combination treatment as reflected in the EFNS guidelines for the diagnosis and management of AD (Hort et al. 2010). However, patients included in the study published by Porsteinsson et al. (2008) had a mild to moderate degree of AD compared to moderate to severe disease severity in the study published by Tariot et al. (2004). It should be noted that memantine is not approved for the treatment of mild AD, neither in the European Union nor in the USA or Japan. In order to address the differences in study populations of these two studies (Tariot et al. 2004; Porsteinsson et al. 2008), a post hoc meta-analysis was performed. In this analysis, in which patients with mild AD were excluded, significant benefits of the combination treatment compared to donepezil monotherapy were seen for patients with moderate to severe AD (Atri et al. 2013). This analysis provides further support that the combination treatment is the most effective in moderate to severe AD patients.

A randomised, double-blind controlled clinical study with 677 moderate to severe AD patients which investigated an extended release formulation of memantine 28 mg in combination with an AChEI compared to AChEI alone has, again, shown statistically significant treatment effects favouring combination treatment (Grossberg et al. 2008; Bassil et al. 2010).

The most recent randomised, controlled clinical study investigated what happens to the patients with moderate to severe AD treated with donepezil when: (a) memantine was added to the existing donepezil therapy (combination treatment group); (b) existing donepezil monotherapy was continued (donepezil monotherapy treatment group); (c) memantine was introduced to the patients and donepezil was discontinued (memantine monotherapy treatment group) and (d) donepezil was discontinued without introduction of memantine (placebo treatment group) (Howard et al. 2012). Due to recruitment difficulties, the initially calculated sample size of 800 patients had to be reduced to 295 recruited patients. In addition, there was a high and disproportionate treatment discontinuation rate (approximately 60 %), leaving only 20 patients in the placebo arm and 38 patients in the combination arm at end of study (week 52). Yet, despite these limitations, the best treatment effects, seen in both primary efficacy parameters (cognition and activities of daily living), were achieved in the combination treatment group at week 30 and were still present at week 52 (although no longer statistically significant for the given small sample size).

Regarding disease progression, pooling clinical data from two randomised controlled studies showed that combination therapy could significantly reduce the occurrence of marked clinical worsening in patients with moderate to severe AD, as compared with AChEI monotherapy (Wilkinson and Andersen 2007; Atri et al. 2013). Consistent with this, observational studies found that combination treatment appeared to slow cognitive and functional decline in the long term (modelled progression over 4 years vs AChEI treatment alone) (Atri et al. 2008), and may delay time to nursing home admission (Lopez et al. 2009). Interestingly, an open-label study also showed that the majority of patients with moderately severe AD, who had continued cognitive decline with AChEI monotherapy, responded positively (stable/improved Mini-Mental State Examination (MMSE) score) upon addition of memantine (Dantoine et al. 2006).

Based on the results from the available clinical studies, the authors of a recent clinical review concluded that the combination therapy for AD seems to be safe, well tolerated and may represent the current gold standard for moderate to severe AD (Patel and Grossberg 2011). In another recent clinical review Schmidtke et al*. (* 2011) state that combination therapy with memantine and an AChEI should be implemented when the patient progresses from *mild* to *moderate* AD. This has been shown in a recent cohort study, in which 686 patients with mild to moderate AD from 16 specialised clinics in France were followed for 4 years (Gillette-Guyonnet et al. 2011). Whereas 89 % of patients were treated with AChEI monotherapy at baseline, 26 % used memantine/AChEI combination therapy by year 4. This is also in accordance with the approved labels, since in Europe AChEIs are approved for the treatment of patients with *mild* AD, whereas both drug groups (AChEIs and memantine) are approved for moderate AD.

Combination therapy is already considered in the current clinical practice of AD treatment, but the frequency of its use differ between countries (Calabrese et al. 2007; Vidal et al. 2008). Variations in the prescription rates of combination therapy between different countries reflect real-life routine clinical practice as well as differences in the reimbursement status in each country. Combination treatment was found to be cost-effective compared with the use of an AChEI alone (Lachaine et al. 2011; Weycker et al. 2007) and clinical studies clearly provide evidence for its usefulness. However, the major stumbling blocks hindering the recommendation of combination therapy for even more AD patients seem to be cost and the therapeutically nihilistic attitude among many practitioners that current AD therapies are not worth prescribing (Patel and Grossberg 2011). As a consequence, a large number of AD patients might be deprived of the currently best possible treatment. This is of special concern for a devastating condition like AD, for which no cure exists and for which combining existing treatments with different modes of action is a valid approach towards treatment optimisation.

In summary, preclinical data confirm that the combination of two existing treatment mechanisms—memantine and an AChEI—may be a useful approach for the management of AD. The two drugs target different (although interconnected) pathological pathways, and it has been proposed that their complementary activity may produce greater effects than either drug alone. This theory has been borne out by results from the currently available clinical studies of combination therapy, which have demonstrated significant efficacy (beyond that of AChEI monotherapy) and good tolerability in patients with moderate to severe AD.
